# Autoantibody to MDM2: A potential serological marker of primary Sjogren's syndrome

**DOI:** 10.18632/oncotarget.14882

**Published:** 2017-01-28

**Authors:** Yuan Liu, Xining Liao, Ying Wang, Shiju Chen, Yuechi Sun, Qingyan Lin, Guixiu Shi

**Affiliations:** ^1^ Department of Rheumatology and Clinical Immunology, The First Affiliated Hospital of Xiamen University, Xiamen, China; ^2^ Medical College, Xiamen University, Xiamen, Fujian, China; ^3^ Department of Endocrinology, The ChengGong Hospital Affiliated to Xiamen University, Xiamen, Fujian, China

**Keywords:** primary Sjogrens syndrome, anti-MDM2 autoantibody, biomarker, Immunology and Microbiology Section, Immune response, Immunity

## Abstract

**Introduction:**

Primary Sjogrens Syndrome (pSS) is one of the autoimmune diseases characterized by polyclonal autoantibody production. The human homologue of the mouse double minute 2 (MDM2) is an important negative regulator of p53. Our previous study indicated that autoantibody to MDM2 can be detected in systemic lupus erythematosus patients. The purpose of this study is to study anti-MDM2 autoantibody in pSS patients.

**Methods:**

Anti-MDM2 autoantibody in sera from 100 pSS patients and 74 normal controls was investigated by ELISA. Positive samples were further confirmed by western blotting. Expression of MDM2 in labial gland tissue from pSS patients and normal controls was checked by immunohistochemistry. The difference in clinical characteristics and laboratory findings between anti-MDM2 positive and anti-MDM2 negative pSS patients was analyzed.

**Results:**

The presence of anti-MDM2 autoantibody in pSS patients was 21.0%, significantly higher than normal controls (5.40%). MDM2 was overexpressed in labial gland from pSS patients. pSS patients with positive anti-MDM2 were characterized by longer disease duration and more lymphocytes focal gathering in labial gland. Prevalence of anemia, thrombocytopenia and anti-SSB was significantly higher in pSS patients with anti-MDM2 autoantibody. Titer of anit-MDM2 was negatively associated with hemoglobin level, platelet count, complement 3 level and complement 4 level, positively associated with European Sjogrens syndrome disease activity index (ESSDAI) and level of IgG.

**Conclusions:**

Anti-MDM2 autoantibody may be used as a potential serological biomarker in pSS disease activity evaluation. Study on the role of anti-MDM2 or MDM2 in pSS may help us know the pathogenesis mechanism of pSS better.

## INTRODUCTION

Primary Sjogren's Syndrome (pSS) is a chronic autoimmune systemic disease, characterized by polyclonal autoantibody production, lymphoplasmocytic infiltration, progressive destruction of salivary and lachrymal glands, and fibrosis of the glandular tissue, which leads to ocular and mouth dryness [1].

Autoantibodies can be used as biomarkers for a variety of diseases, particularly in autoimmune diseases. The detection of autoantibodies against intracellular autoantigens can be a useful tool in diagnosis, clinical classification and prognosis evaluation. Furthermore, the research on biological function of autoantibody or its antigens may help us understanding the pathogenesis of autoimmune diseases, and thus may provide new idea for developing therapeutic strategies.

Several autoantibodies have been found in pSS patients. Anti-SSA and anti-SSB autoantibodies are two of the most well studied autoantibodies, with presence of about 52%-67% and 49% in pSS respectively [2, 3]. Anti-SSA and anti-SSB autoantibodies are now used as important parameters in the diagnosis criteria of pSS from ACR (American College of Rheumatology) and EULAR (European League Against Rheumatism) [4]. However, an obvious limitation existed in anti-SSA and anti-SSB in early diagnosis, disease activity evaluation and prediction of prognosis in pSS [5, 6]. Therefore, more new autoantibodies are still needed to be identified in order for better management and understanding of pathogenesis of pSS [7].

The human homologue of the mouse double minute 2 (MDM2), is also known as E3 ubiquitin-protein ligase [8]. Previous studies indicated that MDM2 might degrade several central cell cycle regulators such as p53 and retinoblastoma (Rb) protein [8, 9]. Further studies showed that MDM2 could elicit a functional autologous immune response in human [10]. Those results indicated that MDM2 and anti-MDM2 antibody might be involved in the development of autoimmunity. One of our previous studies showed that autoantibody to MDM2 can be detected in systemic lupus erythematosus (SLE) [11]. The prevalence of anti-MDM2 in other autoimmune diseases such as pSS patients was still unclear.

In this study, we investigated the presence of autoantibody to MDM2 in sera of pSS patients and normal human sera (NHS). We found that prevalence of autoantibody to MDM2 was significant higher in pSS patients and MDM2 was overexpressed in labial gland of pSS patients. pSS patients with anti-MDM2 autoantibody were presented with clinical characteristics associated with high disease activity. Our study indicated that anti-MDM2 might be used as potential serological biomarker in pSS disease activity evaluation, and study on the role of anti-MDM2 or MDM2 in pSS may help us know the pathogenesis mechanism of pSS better.

## RESULTS

### Prevalence of autoantibody to MDM2 in pSS patients

**Figure 1:**
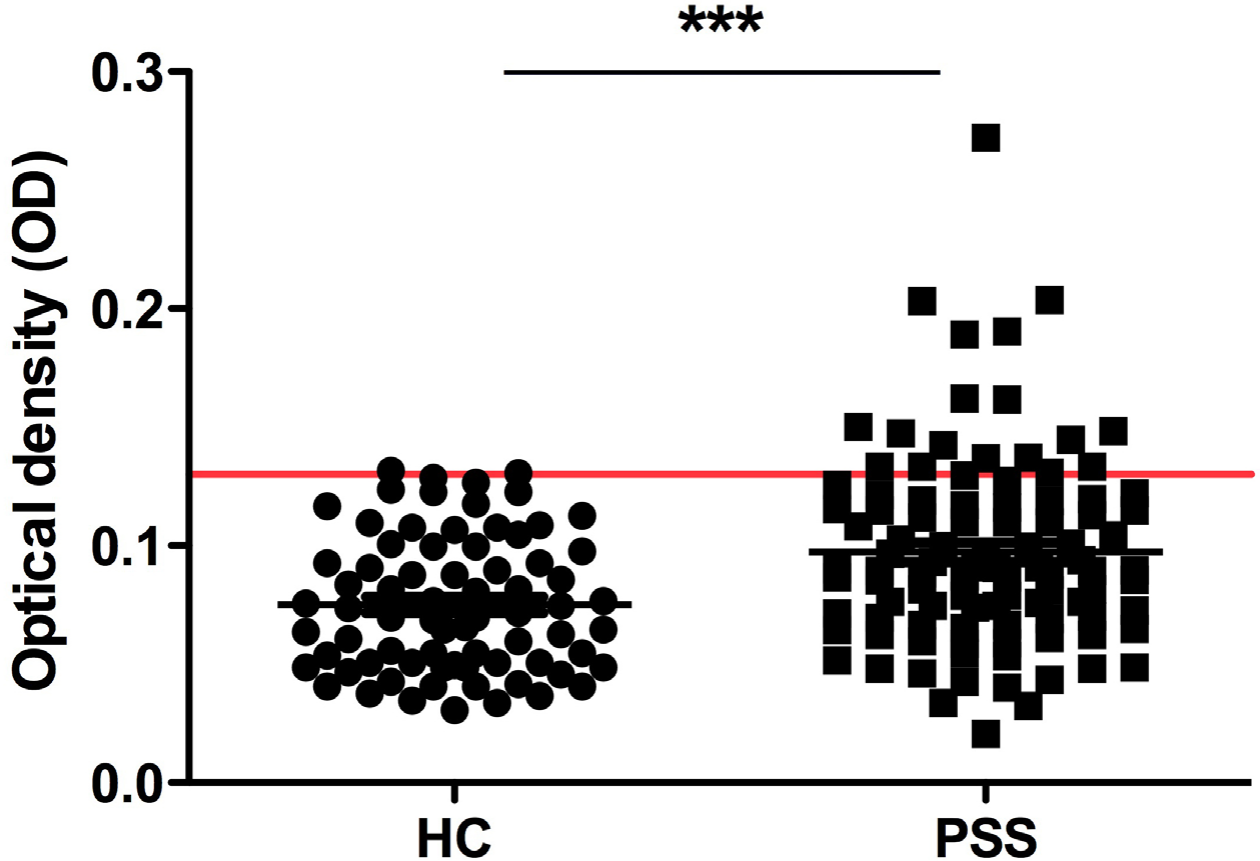
Titer of anti-MDM2 autoantibody by ELISA The range of antibody titers to MDM2 was expressed as optical density (OD) obtained from ELISA. The mean + 2SD of NHS was shown in relationship to all serum samples. Titer of anti-MDM2 in pSS serum was much higher than that in NHS (P < 0.0001). The cutoff value line for positive samples is indicated in the figure.

Serum level of autoantibody to MDM2 in 100 pSS patients and 74 normal human sera (NHS) was determined by ELISA. The mean titer of autoantibody to MDM2 in pSS patients was significantly higher than that in NHS (Figure 1).

Subsequently, we used the mean OD value plus 2SD of NHS as the cut-off value to determine the frequency of anti-MDM2 autoantibody positive sera in pSS and heath control group. The prevalence of anti-MDM2 autoantibody in pSS patients group (21.0%) was significantly higher than NHS group (5.4%)(Table 1).

**Table 1:** Frequency of autoantibody against MDM2 in human sera by ELISA

	**Number**	**Anti-MDM2 (+)**	**Frequency**
NHS	74	4	5.4%
pSS	100	21	21.0%*

NHS: normal human sera; pSS: primary Sjogren's syndrome; *P < 0.05

**Figure 2:**
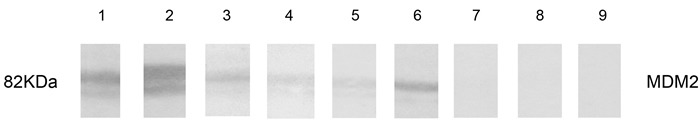
Western blotting analysis with representative sera in ELISA Lanes 1-6: six representative pSS sera that were anit-MDM2 positive in ELISA and also had strong reactivity with MDM2 recombinant protein in western blotting analysis. Lanes 7-9: three randomly selected anti-MDM2 negative NHS by ELISA had negative reactivity with MDM2 recombinant protein.

In order to confirm the presence of anti-MDM2 in pSS patients, anti-MDM2 autoantibody positive sera determined by ELISA were further confirmed by western blotting. Results from Western blotting demonstrated that the anti-MDM2 autoantibody positive serum determined by ELISA also had reactivity with MDM2 recombinant protein, which was shown on Figure 2.

### MDM2 was overexpressed in labial gland in pSS patients

**Figure 3:**
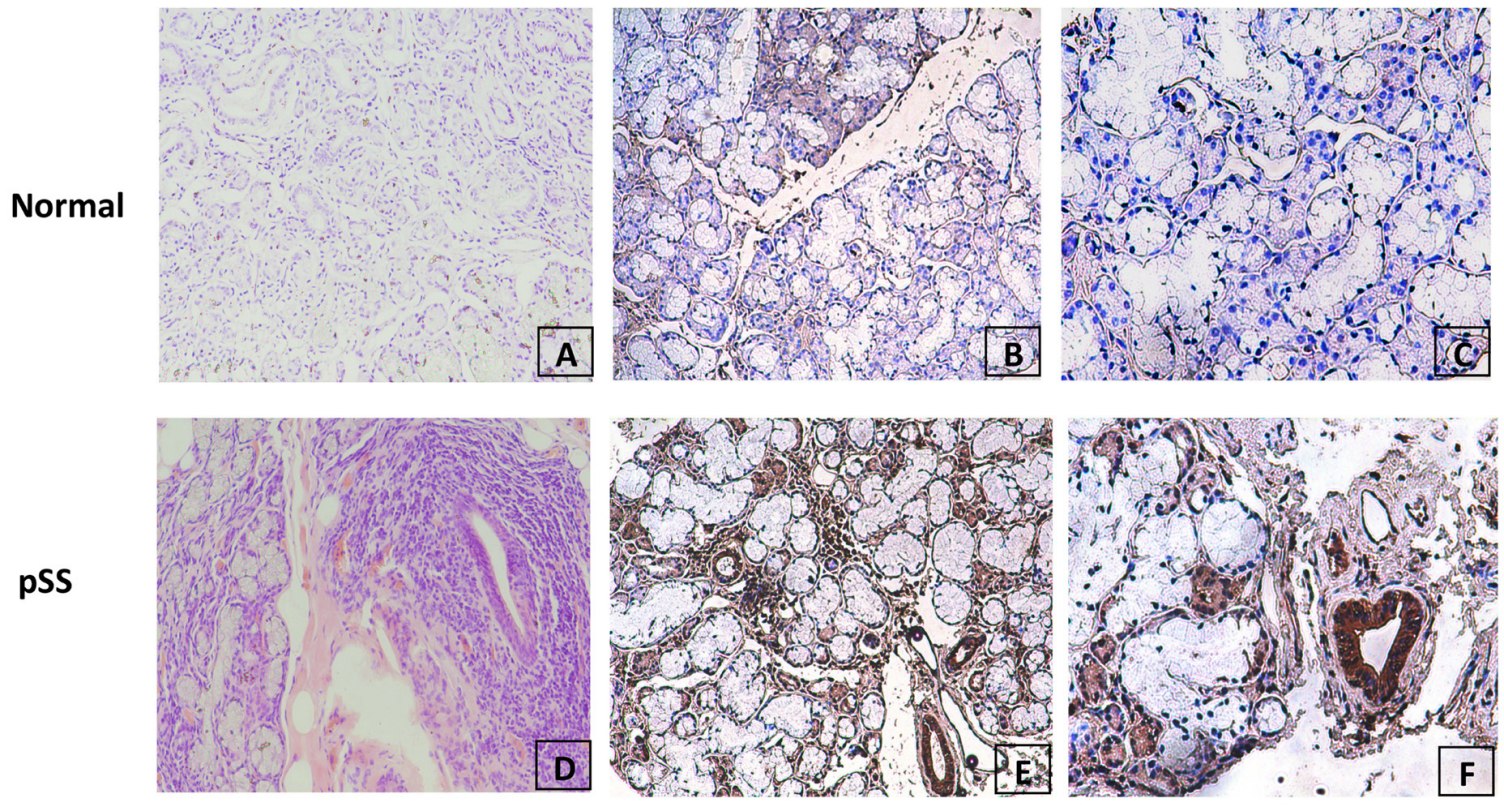
Evaluation of MDM2 protein expression in normal and pSS labial gland tissue by immunohistochemistry **A**. H&E staining of normal labial gland; **B.** & **C**. negative staining of MDM2 expression in representative normal labial gland tissue at 100× and 200× magnification respectively; **D**. H&E staining of pSS labial gland; (E&F) strong positive staining of MDM2 expression in representative pSS labial gland tissue at 100× and 200× magnification respectively.

We further examined the expression of MDM2 in labial gland tissue from 15 pSS patients and 9 controls (patients without lymphocyte infiltration focal in labial gland and can not be diagnosed as SS) by immunohistochemistry. The polyclonal anti-MDM2 antibody was used as primary antibody to detect the expression of MDM2 in labial gland tissues. We detected MDM2 expression in 11 of 15 (73.3%) in pSS patients’ labial gland and 2 of 7 (22.2%) in controls’ labial gland (Figure 3). The increased frequency of MDM2 expression in pSS labial glands was statistically significant (P=0.033), indicated that MDM2 was overexpressed in pSS labial gland, and MDM2 might be involved in pathogenesis of pSS.

### Clinical characteristics of pSS patients with positive anti-MDM2 autoantibody

In order to figure out the clinical significance of anti-MDM2 autoantibody in pSS patients, we further analyzed the difference between clinical characteristics and laboratory findings between anti-MDM2 positive and anti-MDM2 negative pSS patients.

Anti-MDM2 positive and anti-MDM2 negative pSS patients’ clinical characteristics and laboratory findings were shown in Table 2, 3. The disease duration (months) was significantly longer in pSS patients with anti-MDM2 autoantibody, and more lymphocytes focal were found gathering in labial gland of pSS patients with positive anti-MDM2 autoantibody.

**Table 2:** Comparison of clinical characteristics between pSS patients with positive anti-MDM2 autoantibody and pSS patients without anti-MDM2 autoantibody

	**pSS patients with anti-MDM2(+)**	**pSS patients with anti-MDM2(-)**	***p*** **value**
Subjects (n)	21	79	
Age (years)	45.43±3.17	46.08±1.68	0.859
Sex (Female)	18/21 (85.7%)	73/79 (92.4%)	0.392
Disease duration (months)	55.14±12.88*	27.61±5.63	0.034
Dry mouth(n)	14/21 (66.7%)	46/79 (58.2%)	0.483
Dry eyes (n)	13/21 (61.9%)	48/79 (60.8%)	0.924
Schirmer's Test (+) (n)	15/21 (71.4%)	48/79 (60.8%)	0.368
Salivary gland destruction (n)	12/21 (57.1%)	33/79 (41.8%)	0.208
Labial gland lymphocytes focal gathering	1.38±0.31*	0.71±0.12	0.019
Anemia (%) HB<120 g/L (M), HB<110 g/L (F)	10/21 (47.6%)*	13/79 (16.5%)	0.007
Aleucocytosis(%) WBC<4.0×109/L	6/21 (28.6%)	9/79 (11.4%)	0.080
Thrombocytopenia (%) PLT<100×109/L	5/21 (23.8%)*	5/79 (6.3%)	0.032

Date was presented as mean ± SEM or n (%), *, *P* < 0.05.

**Table 3:** Comparison of laboratory findings between pSS patients with positive anti-MDM2 autoantibody and pSS patients without anti-MDM2 autoantibody

	**pSS patients with anti-MDM2 (+)**	**pSS patients with anti-MDM2 (-)**	***p*** **value**
Subjects (n)	21	79	
Increased C-reactive protein (%) >3mg/L	7/21 (33.3%)	28/79 (35.4%)	0.857
Positive ANA (%)	18/21 (85.7%)	50/79 (63.3%)	0.050
Positive anti-SSA antibody (%)	17/21 (81.0%)	47/79 (59.5%)	0.069
Positive anti-SSB antibody (%)	10/21* (47.6%)	19/79 (24.1%)	0.034
Increased IgG (%) > 15.6 g/L	15/21*(71.4%)	32/79(40.5%)	0.012
Low C3 (%) (< 0.9 g/L)	12/21 (57.1%)	31/79(39.2%)	0.141
Low C4 (%) (< 0.1 g/L)	5/21 (23.8%)	6/79(7.6%)	0.050

Date was presented as mean ± SEM or n (%), *, *P* < 0.05.

Prevalence of anemia and thrombocytopenia in pSS patients with positive anti-MDM2 was as high as 47.6% and 23.8% respectively, significant higher than pSS patients without anti-MDM2 autoantibody. Prevalence of aleucocytosis, dry mouth and salivary gland destruction was higher in pSS patients with positive anti-MDM2, though the difference was not significant.

Prevalence of anti-SSB in pSS patients with positive anti-MDM2 was 47.6%, significant higher than pSS patients without anti-MDM2 autoantibody. Increased level of IgG was also more common in pSS patients with positive anti-MDM2. Although prevalence of positive anti-nuclear antibody (ANA), anti-SSA, low level of complement 3 (C3) and low level of complement 4 (C4) were higher in pSS patients with positive anti-MDM2, the difference was not significant. No significant difference in other laboratory findings including C-reactive protein and anti-SSA was detected between anti-MDM2 positive and anti-MDM2 negative pSS patients.

There were 18 pSS patients who were negative with both anti-SSA and SSB in our study, and 2 of them (11.1%) were positive with anti-MDM2. This implied that anti-MDM2 might be used as a complementary biomarker with anti-SSA or anti-SSB in diagnosis of pSS, though the samples were relatively small.

Titer of anti-MDM2 autoantibody was positive correlated with European Sjogren's syndrome disease activity index (ESSDAI), negatively associated with hemoglobin level, platelet count, complement 3 (C3) and complement 4 (C4)

We further analyzed the correlation of titer of anti-MDM2 and European Sjogren's syndrome disease activity index (ESSDAI) [12], complement 3 (C3), complement 4 (C4), immunoglobulin and other laboratory findings including level of platelet (PLT), hemoglobin (HB) and white blood cell count (WBC).

**Figure 4:**
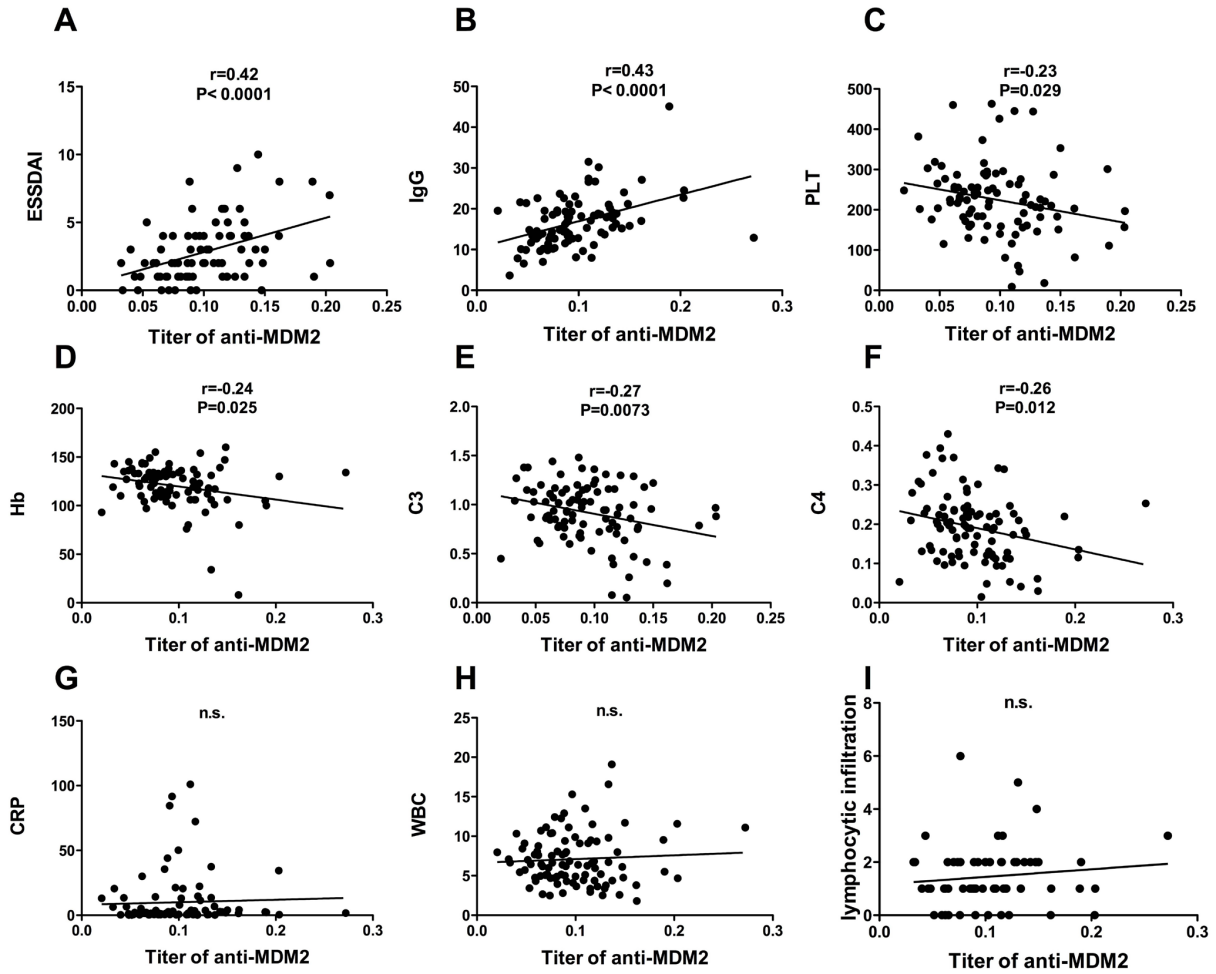
Clinical significance of anit-MDM2 autoantibody in pSS patients Association of titer of anit-MDM2 and ESSDAI **A**.; IgG, g/L **B**.; platelet count, ×109/L **C**.; hemoglobin, g/L **D**.; complement C3, g/L **E**.; complement C4, g/L **F**.; c- reactive protein, mg/L **G**.; white blood cell count, ×109/L **H**. and lymphocytic infiltration **I**. were determined by using pearson's correlation coefficients, significant positive association was found between titer of anit-MDM2 and ESSDAI and level of IgG, significant negative association was found between titer of anti-MDM2 hemoglobin count, platelet count, complement 3 (C3) and complement 4 (C4).

As shown in Figure 4, titer of anti-MDM2 autoantibody was significantly positively correlated with ESSDAI and level of IgG. A significant negative correlation was found between titer of anti-MDM2 and level of PLT, HB, C3 and C4. No significant correlation was found between titer of anti-MDM2 and WBC, CRP and lymphocytic infiltration in labial salivary gland.

## DISCUSSION

Our study showed that anti-MDM2 autoantibody was presented in 21.0% pSS patients, significantly higher than health controls (5.4%). Anti-SSA and anti-SSB autoantibodies were used as important parameters in the diagnosis of pSS [4]. However, not every pSS patients were positive with anti-SSA and/or anti-SSB, which lead to difficulties in pSS diagnosis. Several studies have found new biomarkers such as anti-SP1 may be used as complementary biomarker with anti-SSA or anti-SSB, which greatly help us in the early diagnosis of pSS [13]. In our study, we found that 11.1% (2/18) pSS patients negative with both anti-SSA and anti-SSB were presented with anti-MDM2, indicating anti-MDM2 might be used as a complementary biomarker with anti-SSA or anti-SSB.

Though anti-SSA and anti-SSB have been used as important biomarker in pSS diagnosis for a long time, no clear evidence showed that they were associated with disease activity of pSS. Our study found pSS patients with positive anti-MDM2 were characterized by longer disease duration and more lymphocytes focal gathering in labial gland. Prevalence of anemia, thrombocytopenia and anti-SSB were significantly higher in pSS patients with anti-MDM2 autoantibody. Titer of anti-MDM2 was negatively associated with hemoglobin level, platelet count, complement 3 (C3) level and complement 4 (C4) level, positively associated with European Sjogren's syndrome disease activity index (ESSDAI) and level of IgG. Our data suggest that anti-MDM2 autoantibody might be used as a potential serological biomarker for disease activity evaluation of pSS.

Besides the strong association of anti-MDM2 with clinical characteristics related to high disease activity of pSS, we also found MDM2 was overexpressed in labial gland of pSS patients, indicating anti-MDM2 or MDM2 might be involved in pathogenesis of pSS. The MDM2 protein (also known in humans as Hdm2) may be the strongest inhibitor of apoptosis found so far, and it was described as one of the tumor associated antigens (TAA) as MDM2 was overexpressed in several kinds of tumor [14]. MDM2 was an important negative regulator of p53. Several studies have proved the negative regulation of p53 on autoimmunity [15, 16]. The effect of MDM2 on the immune regulation may be speculated by its inhibition on p53 [17]. The direct role of MDM2 in immune regulation has been showed by several studies in recent years. Gasparini et al. showed that inhibitor of MDM2 can promote dendritic cell-induced T cell proliferation[18]. Mulay et al. found that MDM2 was required to induce mRNA expression and secretion of NF-ΚB-dependent cytokines upon Toll-like receptor stimulation[19]. So far, there is no clear evidence indicated that MDM2 was involved the pathogenesis of pSS. However, several studies have shown MDM2 may promote the pathogenesis of SLE[20]. The specific mechanisms of how anti-MDM2 or MDM2 was involved in pSS pathogenesis still needed to be investigated.

Our study was the first to demonstrate the presence of anti-MDM2 in pSS patients. However, our present study also have some oblivious limitation, such as the number of patients included in our study was relatively small, especially those pSS patients negative with both anti-SSA and anti-SSB. Clinical significance of anti-MDM2 in pSS patients still need to be investigated in large number of pSS patients with detailed clinical information.

In conclusion, our study reported high prevalence of anti-MDM2 in pSS patients. Anti-MDM2 may be used as a complementary biomarker with anti-SSA or anti-SSB to assist the diagnosis of pSS, and a potential serological biomarker for disease activity evaluation of pSS. Further study on how anti-MDM2 or MDM2 was involved in pSS may help us understand pathogenesis mechanism of pSS better.

## MATERIALS AND METHODS

### Sera and patients

In the current study, sera from 74 normal human (NHS) and 100 pSS patients were examined. These sera were obtained from the serum bank of The First Affiliated Hospital of Xiamen University. The diagnosis of pSS was established according to the American College of Rheumatology criteria [21]. The medical ethic committee of The First Affiliated Hospital of Xiamen University has approved this study.

Basic and clinical data in 100 pSS patients including age, sex, detailed patient history, clinical manifestations, laboratory findings, and treatment strategy were collected from patients’ medical records simultaneously with sera obtained.

### Expression and purification of recombinant MDM2

Recombinant protein of MDM2 was derived from previous studies [22]. MDM2 cDNAs was subcloned into pET28a vector producing fusion proteins with NH-terminal 6x histidine and T7 epitope tags. Recombinant protein was further expressed in E. coli BL21 (DE3) and then purified using nickel column chromatography (Qiagen, Valencia, USA). Reactivity of the purified recombinant protein have been analyzed by electrophoresis on SDS- PAGE and determined with polyclonal anti-MDM2 antibody (GeneTex, Irvine, USA).

### Enzyme-linked immunosorbent assay

We used the standard protocol established by our previous research for ELISA [23]. First, we used purified recombinant proteins MDM2 that were diluted in phosphate-buffered saline (PBS) to a final concentration of 0.5 μg/ml and coated onto a 96-well microtiter plate overnight at 4°C. We blocked the antigen-coated wells with BSA at room temperature for 2 hours. Then, 1:100 diluted human sera samples were incubated with the antigen-coated wells for 2 hours at room temperature. We used peroxidase-conjugated rabbit anti-human IgG (EUROIMMUN) as a secondary antibody and the substrate 3,3’, 5,5’-tetramethylbenzidine (TMB)/hydrogen peroxide (H_2_O_2_) (EUROIMMUN) as the detecting reagent. The average OD value of each well was read at 450 and 620 nm, and then used for data analysis. Each sera sample was tested in duplicate. We designated a positive sample using the cutoff value which was the mean OD of 74 normal human sera (NHS) + 2SD.

### Western blotting

Sera with positive anti-MDM2 determined by ELISA were further confirmed by western blotting. In brief, the recombinant MDM2 protein was electrophoresed by 10% SDS-PAGE and then transferred to a nitrocellulose membrane. The nitrocellulose membrane was cut in strips and blocked each strips in PBS containing 3% nonfat milk and 0.2% Tween-20 for 1 hour at room temperature. Subsequently, the nitrocellulose strips were incubated overnight with 1:200 dilutions of serum samples at 4°C. Finally, the strips incubated with peroxidase-conjugated rabbit anti-human IgG (EUROIMMUN) which applied as secondary antibody for 1 hour at room temperature. Positive signals were captured by autoradiography using chemiluminescence (BIO RAD) according to the manufacturer's instructions.

### Immunohistochemistry with labial gland tissue slides

Labial gland from pSS patients and health controls was obtained from tissue bank of The First Affiliated Hospital of Xiamen University. Expression of the MDM2 protein was detected in those tissues. Tissue slides were deparaffinized with xylene and dehydrated with ethanol. Antigen retrieval was performed by microwave heating methods in Trilogy pretreatment solution for 20 min. Avidin/biotin blocking solution was used to prevent nonspecific binding of antibodies. The sections were incubated with polyclonal anti-MDM2 antibody (1 : 50 dilution) for overnight at 4°C. HRP Detection System (HRP streptavidin label and polyvalent biotinylated link) and DAB Substrate Kit were used as detecting reagents. After counterstaining with hematoxylin, the sections were dehydrated and mounted. The slides were observed by light microscopy (Leica DM1000, Germany).

Stained sections were semi-quantitated using methods described by Jian-Min Tang[24]. Briefly, cells showing cytoplasm and/or nucleus staining were judged as positive. Five high-power fields in light microscope were selected randomly. The average percentage of cell staining positively was calculated in each field among 200 cells counted. The average percentage of positive cell was designated as 0 when no cell stained, 1 when10—20% of cells stained, 2 when 20—50% of cells stained, and 3 when >50% of cells stained. The intensity of cell staining positively was categorized as follows: 0, no appreciable staining in cells; 1, barely detectable staining as compared with stromal elements; 2, readily appreciable brown staining distinctly marking cell cytoplasm and/or nucleus; and 3, dark brown staining in cells. Scoring was performed by multiplying staining intensity score and average percentage of cell staining positively, ranging from 0 to 9. For purposes of statistical analysis, all cases scoring 2 or less were grouped as negative expression and all cases scoring 3 or greater were grouped as positive expression.

### Statistical analysis

All data were represented as mean ± standard deviation (SD). The <2 test with Fisher's exact test was used to compare the frequency of autoantibody to MDM2 in the sera. Correlation coefficients were calculated using the Spearman rank correlation analysis. Statistical analysis was performed in SPSS19.0 software, and P < 0.05 was considered statistically significant.
